# Why are some neurons spared in neurodegeneration? Cellular subtype identity maintenance as a candidate threshold-setting mechanism

**DOI:** 10.1371/journal.pbio.3004014

**Published:** 2026-10-05

**Authors:** Dong Won Kim

**Affiliations:** 1 Danish Research Institute of Translational Neuroscience (DANDRITE), Nordic EMBL Partnership for Molecular Medicine, Aarhus University, Aarhus, Denmark; 2 Department of Biomedicine, Aarhus University, Aarhus, Denmark; School of Medicine and Health, Technical University, Munich, GERMANY

## Abstract

Neurodegenerative diseases often damage some neuronal populations while sparing closely related neighbors. But instead of asking why some neurons die, should we be asking why some survive? This Essay proposes that a neuron’s capacity to sustain the gene-regulatory machinery that maintains its subtype identity helps set the stress threshold beyond which recovery fails. The initiating insult may be metabolic, proteostatic, inflammatory, or arise from the surrounding tissue, but it is the cell’s capacity for maintenance that will determine where the tipping point lies: above a critical level, the identity program recovers, whereas below it, regulatory loss becomes self-sustaining. Such a framing could have implications for how neuronal vulnerability to neurodegeneration is understood.

## Abstract 2

Why do some neurons die in neurodegenerative disease while closely related neighbors survive? This Essay argues that disease resistance may depend on active maintenance of adult neuronal identity and proposes testable ways to distinguish identity failure from reversible cell-state change.

## Introduction

Neurodegenerative diseases do not uniformly affect the nervous system. In Parkinson’s disease, dopamine neurons in the substantia nigra pars compacta (SNc) are lost extensively at an early stage, whereas many dopamine neurons in the ventral tegmental area (VTA) survive for longer [1–3]. In Alzheimer’s disease, layer II entorhinal cortex neurons and connected cortical regions are affected early on [2,4], and in amyotrophic lateral sclerosis (ALS; also known as motor neuron disease), fast-fatigable motor neurons are among the first to fail [5,6]. These examples illustrate the central problem of selective vulnerability: closely related or neighboring neurons can encounter partly overlapping pathological stressors, yet follow different trajectories [1–7].

Most explanations of selective vulnerability begin with the dying cell. They emphasize calcium burden, axonal architecture, mitochondrial stress, proteostatic load, excitability, exposure to misfolded proteins, inflammation, or synaptic demand [1,2,5–7]. These mechanisms are important and experimentally productive; however, they can fail to answer the complementary question of what enables resistant neurons to persist under the same pressure. One possible answer concerns what it takes for a mature neuron to remain the kind of neuron it is.

A mature neuron does not necessarily retain its identity as a static developmental inheritance. In some systems, subtype identity depends on the continued operation of the gene-regulatory machinery that first helped establish that identity and now maintains its defining features [8–14]. If this maintenance machinery weakens, the neuron may not die immediately. It may first become less precisely itself. In this Essay, I propose that a neuron’s capacity to sustain this machinery helps set the stress threshold beyond which recovery fails. In this view, resistant neurons are not simply exposed to less stress or endowed with a more favorable baseline physiology; they may also preserve the regulatory architecture that defines subtype identity as stress rises (see Box 1 for an example of how this might work with dopamine neurons in the SNc and VTA in Parkinson’s disease). This framework does not assume that identity erosion is the initiating lesion: metabolic burden, axonal architecture, proteostatic failure, inflammation, or loss of niche support could all act upstream, with identity erosion occurring secondarily. In that case, identity erosion might be an early and reliable marker of degeneration without determining vulnerability. The claim I am making is distinct from that possibility, and narrower than a causal-initiation claim: identity-maintenance capacity contributes to where the transition between recoverable and unrecoverable states occurs. This predicts that changing maintenance capacity will shift both the stress level at which function fails and the capacity to restore subtype-defining architecture after stress.

**Box 1. The SNc–VTA puzzle in Parkinson’s disease**

The contrast between substantia nigra pars compacta (SNc) and ventral tegmental area (VTA) dopamine neurons is useful because it is easy to state and hard to explain. Both populations use dopamine and arise from related developmental programs, although they contribute to partly distinct basal ganglia and limbic circuits. However, in Parkinson’s disease, the SNc population, particularly those neurons projecting broadly to the striatum, is much more vulnerable to degeneration than many VTA neurons [1–3]. The VTA is not immune to Parkinsonian pathology, but its relative preservation highlights a key biological question. Why do related dopamine neurons, separated by a short anatomical distance and sharing a broad transmitter identity, differ so sharply in their disease trajectory?

Classic explanations point to features enriched in vulnerable SNc neurons, including large axonal arbors, autonomous pace-making, calcium handling, mitochondrial demand, and proteostatic stress [1,2,7]. The identity-maintenance view suggested in this Essay does not replace these explanations. It proposes that the effects of these stressors also depend on how long a neuron can preserve its subtype-defining regulatory architecture as stress increases. In the threshold model, two populations exposed to comparable insults could reach functional or irreversible failure at different stress levels because their identity-maintenance programs differ in robustness. A resistant neuron is therefore not necessarily exposed to less stress; it may instead retain a coherent regulatory program at a stress level that destabilizes the same program elsewhere.

Framed this way, the puzzle becomes a question of regulatory architecture rather than dopamine. It is therefore important to define what would count as an explanation. The transcription factor Nurr1 is often invoked in this context [22–24], but it is expressed in both SNc and VTA neurons [22,23], and a factor shared by both populations cannot by itself explain their different vulnerabilities. Divergence could instead arise from differences in how such factors are wired, including whether the same nominal maintenance program is organized, buffered, or made redundant in different ways across each population. Another possibility is that the shared network differs in chromatin sensitivity, such that stress more readily disrupts Nurr1 expression, target gene accessibility, or regulatory coupling in SNc neurons. Differential network architecture and differential chromatin sensitivity are distinct explanations and will require different experiments to distinguish them.

A mechanistic basis for this threshold could lie in self-reinforcing regulatory circuits, in which identity-factor activity, enhancer accessibility, and repression of alternative programs stabilize one another. Sufficiently deep disruption could make regulatory loss self-sustaining and produce hysteresis, in which recovery depends on the system’s prior state and may require stress to fall below the level at which disruption was initiated. The framework rests on two claims that should not be conflated. First, at least some mature neuronal identities require continued transcriptional and chromatin-based maintenance. This claim has direct experimental support from perturbations of transcription factors and chromatin regulators in differentiated or adult neurons [8–14,17,18]. Second, differences in the robustness, architecture, or stress sensitivity of these maintenance programs may contribute to selective vulnerability. Evidence for this second claim is substantially more limited and should be treated as a cell-type- and disease-specific hypothesis rather than a universal principle of neurodegeneration. This distinction matters in practice: the first claim establishes that active identity maintenance exists, whereas the second must be tested in individual neuronal populations.

In this Essay, I first review evidence that mature neuronal identity requires continued transcriptional and chromatin-based regulation. I then distinguish reversible state remodeling from identity-maintenance failure, examining evidence involving the transcription factors Nurr1 and Pitx3 and the histone-modifying complex PRC2, and outline observational and interventional tests of causality. I conclude by considering aging, non-cell-autonomous support, therapeutic implications, and comparative evidence from Alzheimer’s disease and ALS.

## Adult neuronal identity is maintained, not merely inherited

A mature neuron can appear stable because it has a transmitter, projection pattern, receptor repertoire, electrophysiological profile, and circuit role. That stability can make identity look like a completed developmental product. Developmental neurobiology, however, has shown that view to be too simple.

In *Caenorhabditis elegans*, terminal selector transcription factors regulate batteries of genes that define mature neuronal features, including neurotransmitter enzymes, receptors, channels, and neuropeptides [9–11]. Several terminal selectors are required not only to establish identity during development but also to maintain it in mature neurons [9,10]. When maintenance fails, neurons can retain broad neuronal character while losing the molecular features that made them a particular neuronal type [11]. Work on the cholinergic motor neuron selector UNC-3 further shows that selector activity is not confined to a fixed set of cell identity markers. UNC-3 maintains classic terminal-identity effectors, changes parts of its target repertoire across the life course, and regulates additional transcriptional, functional, and metabolic programs [15,16]. This distinction matters for interpreting what happens with transcription factors like Nurr1, which is implicated in neuronal vulnerability in Parkinson’s disease (Box 1). A maintenance factor may directly control subtype-defining genes alongside homeostatic or metabolic functions, or homeostatic decline may follow secondarily from identity loss. The two architectures make different predictions about generalization because the relative proportion of fate and non-fate direct targets is likely to vary between factors; for Nurr1, the existing data do not distinguish between them.

Among the better-characterized vertebrate transcription factor examples, Pet-1 and Lmx1b support maintenance of the serotonergic program, whereas FEZF2 illustrates how postmitotic neurons suppress inappropriate alternative identities, although its role in lifelong adult maintenance is less well established [12–14]. Adult neuronal identity can also depend on active repression. Loss of PRC2-mediated gene silencing in differentiated dopaminergic and serotonergic neurons de-represses inappropriate programs and destabilizes subtype-specific expression, whereas PRC2 loss in adult striatal neurons causes early identity disruption followed by progressive degeneration [17,18]. Together, these illustrative examples indicate that mature identity may require both continued expression of subtype-defining programs and continued repression of inappropriate ones, with consequences ranging from functional disruption to degeneration.

A major limitation here is that the relevant maintenance circuitry remains poorly defined for most vertebrate neuronal subtypes. In many cases, the required transcription factors are unknown, their direct targets have not been distinguished from downstream effects, and the enhancing and repressive chromatin mechanisms that preserve adult identity have not been mapped. Regulatory redundancy may also mean that one subtype can compensate for the partial loss of a regulatory node while another cannot. This knowledge gap is not merely technical: without a reference model of how neuronal identity is actively maintained in healthy cells, it may be difficult to determine whether a disease-associated molecular change reflects failure of that program or a reversible response within it.

The neuronal case also differs from some non-neuronal systems. Prox1 is required for the developmental acquisition of lymphatic endothelial cell identity, and its conditional loss from differentiated lymphatic endothelial cells can reprogram them towards a blood vessel endothelial phenotype [19,20], whereas loss of Dmrt1 in adult Sertoli cells can produce a coherent conversion towards a granulosa cell program [21]. Comparable wholesale transdifferentiation into a neighboring mature cell type has not been demonstrated after maintenance factor loss in fully differentiated neurons. One possibility is that highly specialized postmitotic neurons lack an accessible and viable alternative fate into which they can convert. Under this interpretation, neuronal identity-maintenance failure would instead appear as partial loss of subtype features, fragmentary expression of normally excluded programs, functional deterioration, or cell death. This possibility remains speculative, but it makes a testable prediction: mixed identity in degenerating neurons should usually be incomplete and modular rather than a clean switch from one mature neuronal type to another.

These cases do not imply that all adult neurons use the same maintenance logic or that developmental and adult regulatory networks are identical. Rather, they indicate that some mature neuronal identities require the persistent or redeployed activity of developmental regulators and chromatin mechanisms, but the architecture, robustness, and functional consequences of these maintenance programs are likely to differ among neuronal populations.

## State remodeling is not the same as identity-maintenance failure

Neurodegeneration involves many transcriptional state changes. The useful distinction is between reversible cell-state remodeling, in which a neuron responds to stress while preserving the regulatory constraints that define its type, and identity-maintenance failure, in which those constraints begin to erode. Observing identity erosion before neuronal death can help to place it within the disease sequence, but temporal precedence alone cannot show that it causes vulnerability. Establishing mechanism requires bidirectional interventions that weaken maintenance programs in relatively resistant neurons or strengthen them in vulnerable neurons, followed by testing across controlled levels of stress exposure.

A neuron changes state whenever it responds to activity, sleep and wakefulness, metabolic challenge, inflammation, injury, or disease. A stressed dopamine neuron may induce heat shock genes, alter mitochondrial transcripts, or activate an unfolded protein response while remaining recognizably dopaminergic. In such cases, the cell’s state changes while the regulatory architecture of the cell type remains largely intact. Identity-maintenance failure is different because the maintenance program itself weakens. Identity transcription factor activity declines or becomes uncoupled from its expected targets, subtype-defining gene modules lose coherence, a coordinated subset of identity enhancers loses accessibility, and fragments of normally excluded programs may appear. The transcriptome does not merely shift; the constraints that stabilize the neuron’s subtype identity become less effective. Fig 1 summarizes this distinction, and Table 1 lists practical readouts that can separate the two processes in single-cell and spatial datasets.

**Table 1:** Distinguishing reversible state remodeling from identity-maintenance failure.

Feature	Reversible cell-state remodeling	Identity-maintenance failure
Primary change	Adaptive response to activity, metabolic, inflammatory, or proteostatic challenge	Weakening of the regulatory machinery that stabilizes subtype identity
Identity transcription factors	Expression or activity remains coherent with expected targets	Activity falls or becomes uncoupled from subtype-defining target modules
Chromatin	Stress-response elements may remodel; identity enhancers remain accessible	A coordinated subset of subtype-defining enhancers loses accessibility or identity-factor motif activity
Gene-expression pattern	Stress modules change while terminal subtype modules remain recognizable	Subtype-defining modules drift, decohere, or become variable across cells
Alternative programs	Excluded neighboring-cell programs remain repressed	Partial or fragmentary expression of normally excluded neighboring-cell programs appears
Recovery prediction	Removal of the perturbation permits a return towards the prior identity state	After sufficiently deep disruption, the subtype-defining regulatory architecture fails to return to the range observed in matched recovered controls once the normal recovery period has elapsed. It may also show path-dependent recovery (hysteresis). Selective loss of the most disrupted cells must be excluded.
Interventional prediction	After generic cellular burden is matched, perturbing the putative maintenance node does not shift the stressor dose–response or recovery trajectory more than a non-identity control perturbation	Across graded manipulations spanning partial to near-complete verified disruption of the targeted identity architecture, weakening the node shifts failure to lower stress levels, whereas strengthening it permits recovery at higher stress levels. This shift must exceed that produced by a non-identity perturbation matched on prespecified generic cellular-burden readouts. If no degree of verified architectural disruption shifts vulnerability or recovery beyond this burden-matched control, the mechanism is not supported in that population. A single null manipulation is uninformative.
Required evidence	Stress-associated changes occur without coordinated erosion of the predefined subtype program	A prespecified composite shows disruption in at least three of four domains (identity factor target coupling, identity regulon or terminal effector coherence, identity enhancer accessibility or motif activity, and repression of excluded programs) with each crossing an assay-specific effect-size threshold relative to matched controls

**Fig 1:**
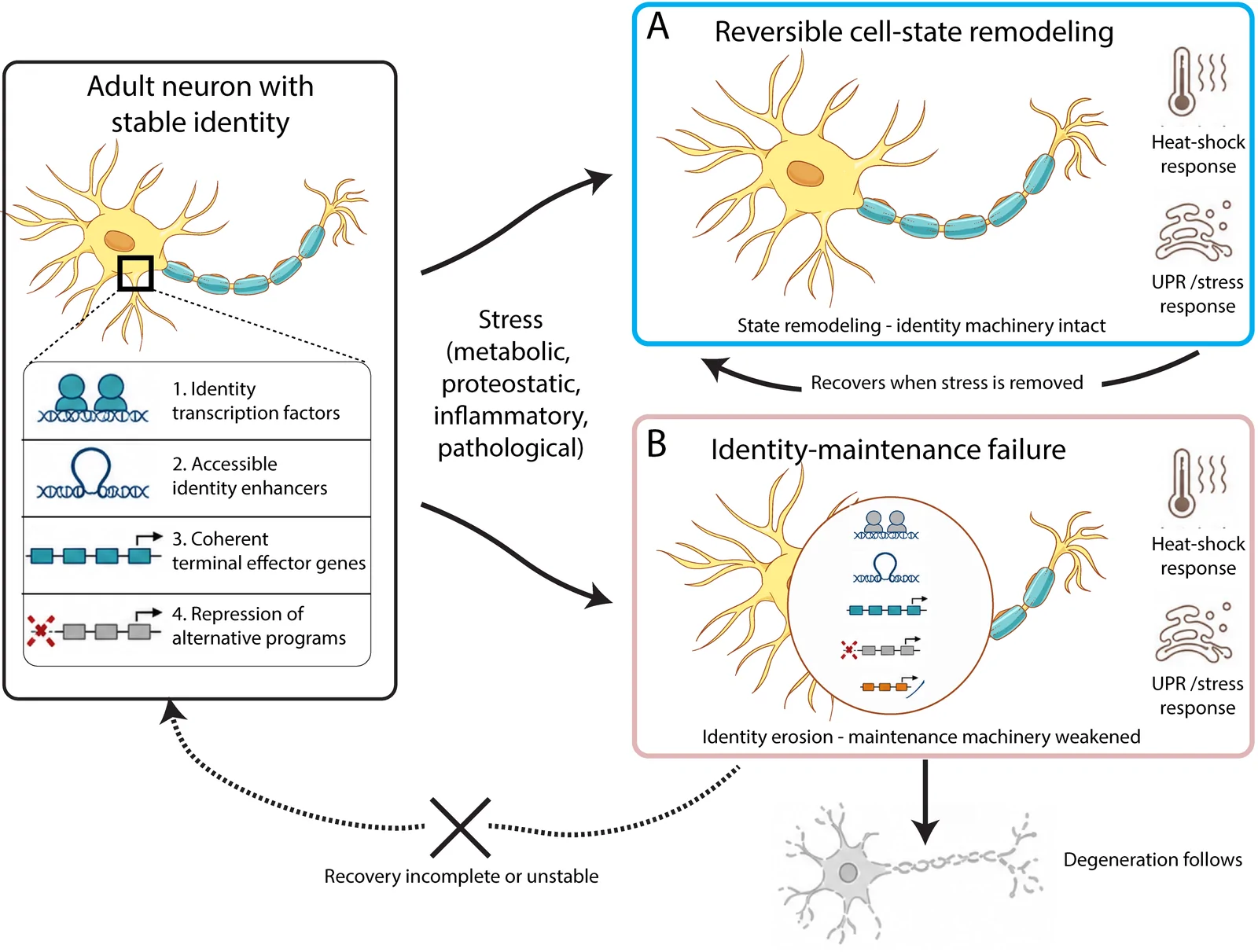
Reversible cell-state remodeling vs. identity-maintenance failure in adult neurons. An adult neuron with stable subtype identity (left) depends on continued operation of four interrelated regulatory components, shown in the inset legend: (1) identity transcription factors; (2) accessible identity enhancers; (3) coherent terminal effector gene modules; and (4) active repression of alternative gene programs belonging to other cell types. Under stress (metabolic, proteostatic, inflammatory, or pathological), the neuron can follow one of two trajectories. **(A)** In reversible cell-state remodeling, the neuron mounts adaptive responses such as the heat-shock response and the unfolded protein response (UPR), while identity transcription factor activity, enhancer accessibility, terminal effector modules, and repression of alternative programs remain intact. When the stress is removed, the neuron returns to its prior identity state. **(B)** In identity-maintenance failure, similar stress responses occur, but the regulatory machinery that stabilizes subtype identity itself weakens: identity transcription factor activity falls, identity enhancers lose accessibility, terminal effector modules decohere, and repression of alternative or neighboring-cell programs (orange) fails, allowing those programs to be expressed. After sustained disruption of this machinery, return to the prior identity state may be incomplete, delayed, or unstable even after the initiating stress is removed (broken recovery arrow), and degeneration may follow. The stress-response components are shown at comparable intensity in both panels to emphasize that the framework concerns identity-regulatory outcomes under matched stress exposure, not differences in stress response itself. Neuron illustration adapted from NIH BioArt Source, “Healthy neuron” (BIOART-000197), Public Domain, Courtesy of NIAID.

Identity-maintenance failure is a mechanistic interpretation rather than a fixed numerical category. State remodeling and maintenance failure may form a continuum, and no universal percentage of lost markers or closed enhancers can define the boundary across neuronal populations. The claim should instead require coordinated disruption of a predefined subtype program relative to healthy cells of the same subtype, relatively resistant populations under comparable pathology, and cells undergoing a matched reversible response. Loss of one marker or a global decline in transcription in low-quality or terminally compromised cells is not sufficient. For operational testing, the analysis plan should prespecify four domains: identity factor target coupling, identity regulon and terminal effector coherence, accessibility or motif activity at identity-associated enhancers, and repression of normally excluded programs. Where all four domains can be measured, classification as candidate identity-maintenance failure should require disruption in at least three, with each crossing a prespecified assay-specific effect-size threshold relative to the matched control distribution. This three-of-four rule is an analytical safeguard against post hoc classification, not a universal biological cutoff. Because the apparent coherence of a subtype depends partly on how finely reference cells are classified, subtype boundaries and regulons should be prespecified before disease analysis, and conclusions should remain stable across biologically plausible clustering resolutions. Where fewer than four domains can be assessed, the classification should be reported as provisional, the unmeasured domains should be stated explicitly, and the available evidence should not be treated as equivalent to a complete composite assessment.

This distinction is experimentally useful because reversible state remodeling should recover when the perturbation is removed, provided the identity machinery remains intact. Identity-maintenance failure should show a different pattern after sufficiently sustained disruption of a maintenance node. Self-reinforcing regulatory circuitry provides a possible mechanism for this difference. If identity-factor activity, enhancer accessibility, terminal-effector expression, and repression of alternative programs reinforce one another, a shallow perturbation may resolve when stress is removed, whereas a deeper perturbation may cross a point at which regulatory loss becomes self-sustaining. Recovery would then show hysteresis: the route back to the original state would differ from the route by which the neuron left it, and removal of the initiating stressor might no longer be sufficient to restore that state. Such hysteresis has not been established as a general property of adult vertebrate neuronal identity networks, but it is a distinctive prediction of the threshold model. Recovery should therefore be assessed relative to the normal kinetics of the neuronal subtype and across perturbations of different intensities and durations, rather than at a single universal time point.

Slow recovery alone would not demonstrate hysteresis. Evidence would require measurements that extend beyond the normal recovery kinetics of the subtype and demonstrate path dependence. For example, the same stress level should produce different stable regulatory states depending on whether it was approached from an intact state or after a stronger perturbation. Selective death of the most disrupted cells must also be excluded because a change in population composition can mimic incomplete recovery. Apparent population-level path dependence could also arise from stable heterogeneity in the stress thresholds of individual neurons rather than from bi-stability within individual cells. Distinguishing these possibilities would require longitudinal single-cell, lineage-resolved, or repeated measurement approaches showing that the same cells occupy different persistent regulatory states depending on their prior trajectory.

In this framework, recovery should not mean global transcriptomic normalization alone; it should mean restoration of the subtype-defining regulatory architecture, including identity regulons, terminal-effector genes, and identity-enhancer accessibility. Identity-maintenance failure also need not involve every subtype-specific enhancer or effector gene. Partial disruption may be consequential when it is coordinated, reproducible, associated with subtype dysfunction, and greater than that observed in matched controls. Observational data can nominate candidate maintenance failures and position them within the sequence of degeneration, but they cannot establish causality. Establishing a causal link will require graded, burden-controlled interventional testing, as set out below.

## Evidence from dopaminergic neurons

One of the clearest vertebrate examples comes from midbrain dopamine neurons and the transcription factor Nurr1. Nurr1 is essential for the development of dopamine neurons and remains expressed in adulthood. Conditional removal of Nurr1 from maturing dopamine neurons causes rapid loss of striatal dopamine and dopaminergic markers, followed by neuronal degeneration, whereas removal in adulthood produces a slower decline in dopamine and subtype-defining features [22]. Subsequent inducible deletion in mature dopamine neurons causes progressive dopamine loss, motor impairment, and dystrophic changes in axons and dendrites [23]. Together, these studies establish a continuing requirement for Nurr1 in the maintenance of adult dopamine neurons. Temporal ordering alone, however, does not establish that identity loss causes later degeneration.

Disease-associated models provide a partial interventional test. In a rat model of α-synuclein overexpression, pathological α-synuclein reduced Nurr1 and Ret expression and disrupted GDNF signaling (which is linked to cell survival) in nigral dopamine neurons [24]. Forced Nurr1 expression restored Ret-dependent signaling and protected neurons despite continued α-synuclein overexpression, showing that manipulation of a maintenance-associated node can alter the response to an ongoing disease-relevant stressor. In dopaminergic cell lines and overexpression models, exposure to the neurotoxin MPP+ and α-synuclein overexpression also reduced Nurr1 protein levels, whereas the transcription factors Pitx3, Foxa2, and Lmx1a were less affected [25]. These factors are components of the broader transcriptional network of midbrain dopamine neurons, so the result suggests that stress can perturb one component without causing the entire network to collapse. However, its relevance to human Parkinson’s disease remains uncertain. Transgenic, viral, and pharmacological enhancement of Nurr1 activity has also been shown to be protective in preclinical Parkinsonian models [24–27]. Together, these experiments establish Nurr1 as a tractable node, the manipulation of which can modify neuronal responses to toxic, inflammatory, and genetic stress. The unresolved question is whether this protection depends on preserving dopaminergic identity.

The central mechanistic ambiguity concerns how Nurr1 confers protection. Two models are possible. In a parallel-output model, Nurr1 regulates dopaminergic identity genes and homeostatic or survival-related genes through partly separable branches. Protection could then result from restored mitochondrial function, axonal integrity, or Ret-dependent trophic signaling, with preservation of identity as a correlated output rather than as the protective mechanism. In an identity-mediated model, weakening the subtype-defining regulatory program destabilizes the functional systems that depend on it, making identity loss part of the causal path to vulnerability.

Nurr1 supports nuclear-encoded mitochondrial genes and dopaminergic fiber integrity [23]. However, transcriptomic analysis has identified genes that are dependent on Nurr1 for expression but did not establish that all mitochondrial genes are direct Nurr1 targets. Existing evidence therefore does not clearly distinguish the parallel-output and identity-mediated models. A discriminating experiment would combine acute, graded Nurr1 perturbation with direct measurements of occupancy or nascent transcription and selective restoration of identity-effector, mitochondrial, or trophic signaling modules. Protection achieved by restoring a homeostatic branch without restoring subtype-defining regulatory coherence would support the parallel-output model. Protection that consistently required preservation of identity regulons, terminal effectors, and identity-associated chromatin under matched stress would support the identity-mediated model. The example of Nurr1 therefore supports the first claim of this Essay: that adult dopaminergic identity and function require active maintenance. The Pitx3 and PRC2 findings extend this principle by showing that related neuronal populations differ in the organization of their maintenance programs and in the consequences of their failure.

As discussed in Box 1, Nurr1 contributes to the dopaminergic program in both SNc and VTA neurons, so its presence alone cannot explain their divergent fates. The more tractable question is whether Nurr1 operates within differently organized maintenance networks in the two populations. During dopaminergic neuron differentiation, Pitx3 potentiates Nurr1-dependent transcription by releasing an SMRT–HDAC corepressor complex from Nurr1 target genes [28]. Dependence on Pitx3 is not uniform across midbrain dopamine neurons: Pitx3-null and aphakia mice show profound disruption of dopamine neuron development in the SNc, whereas the VTA is substantially less affected [29,30]. This asymmetry persists in adulthood. Conditional deletion of Pitx3 from mature dopamine neurons using an inducible DAT-CreERT2 system produced age-dependent motor deficits, striatal dopamine loss, and profound degeneration of SNc neurons, while adjacent VTA neurons were spared [31]. Nurr1 expression remained stable in the surviving VTA neurons, and the authors proposed that an alternative regulatory route may operate in the VTA, potentially supported by uninterrupted Nurr1 expression [31]. Pitx3-deficient SNc neurons also showed early reductions in GDNF and Aldh1a1, together with somatic α-synuclein accumulation [31]. This finding is notable because α-synuclein-mediated Nurr1 downregulation also disrupts GDNF signaling by reducing Ret expression [24]. Two distinct maintenance factors may therefore converge on the same trophic effector system in the same vulnerable population. These findings do not establish that disruption of the Pitx3–Nurr1 network causes selective vulnerability in Parkinson’s disease. The manipulations are genetic ablations rather than disease models, and the direction of the relationship in human pathology remains unknown. Instead, they suggest that closely related dopamine-neuron populations can differ in their dependence on components of a shared identity program. Such differential dependence could set different thresholds for the same physiological burden.

Chromatin-level maintenance provides a second, mechanistically independent example. Conditional deletion of Eed, an obligatory PRC2 component, from differentiated dopaminergic and serotonergic neurons causes progressive de-repression of normally silenced genes and loss of subtype-specific expression, with particularly pronounced identity disruption in SNc neurons but preserved neuronal survival [17]. This separates identity disruption from cell death under the conditions tested: failure of a chromatin-maintenance mechanism can impair SNc molecular identity and motor function while neuronal survival remains preserved. However, Eed deletion was not examined across a graded independent stressor dose–response in that study, so the threshold prediction was not tested. The study therefore does not establish whether PRC2 loss changes the stress level at which dysfunction or degeneration occurs. By contrast, PRC2 loss in striatal neurons causes early identity disruption followed by progressive and fatal degeneration [18]. Together, these studies demonstrate that the consequences of disrupting repressive identity maintenance differ among neuronal populations, but they do not by themselves establish that identity disruption shifts vulnerability to an independent stressor.

An important confound remains. SNc neurons are preferentially vulnerable to 6-OHDA-induced degeneration and show more pronounced identity disruption than VTA neurons after PRC2 loss [17,32]. This convergence identifies the same population as being particularly sensitive to both a toxic stressor (6-OHDA) and a chromatin perturbation (PRC2 loss), but it does not establish that identity-maintenance fragility causes toxicant vulnerability. SNc neurons also have marked physiological liabilities, severe degeneration after adult Pitx3 loss, and pronounced identity disturbance after PRC2 disruption. Identity-maintenance fragility therefore co-varies with established bioenergetic and anatomical liabilities in the principal comparison considered here. An alternative interpretation is that SNc neurons approach failure first for classic reasons, including their large, highly branched axonal arbors, autonomous pacemaking, and associated calcium and mitochondrial demands, and consequently respond more severely to any additional perturbation, including disruption of a transcriptional or chromatin regulator. The available evidence does not exclude this interpretation.

The PRC2 studies establish that molecular identity disruption and cell death can be experimentally dissociated, but they do not show that identity disruption shifts vulnerability to an independent stressor. A more decisive test would separate maintenance capacity from classic liability while incorporating a graded stressor dose–response and a burden-matched non-identity control. This could involve preserving identity architecture without reducing the initiating physiological burden, or lowering calcium, metabolic, or axonal burden without restoring identity architecture and testing whether protection is complete. Another approach would be to identify a population with substantial classic liability that remains resistant because its maintenance circuitry is robust. Until such dissociations are demonstrated, identity maintenance should be treated as a candidate threshold-setting determinant that interacts with classic vulnerability mechanisms, rather than as an independent replacement for them.

## What should identity erosion look like in data?

Identity-maintenance failure should leave signatures that differ from generic stress responses. The central prediction is not simply that vulnerable neurons change their transcriptomes, but that they lose coherence in the regulatory architecture that defines their subtype. The least specific discriminator is transcriptomic identity drift: reduced mean expression and increased cell-to-cell variance of subtype-defining genes, with pan-neuronal features relatively preserved. Drift is useful as an early warning sign, but on its own it can resemble generic transcriptional decoherence in failing cells. It becomes more informative when concentrated in predefined identity regulons rather than spread broadly across the transcriptome, and when it is paired with stronger evidence from chromatin or perturbation-recovery experiments. Additional signatures include uncoupling of identity transcription factors from their expected targets, partial expression of normally excluded neighboring or sibling programs, and spatial blurring towards related local populations. In neurons, such mixed identity is expected to be fragmentary rather than a complete conversion into another mature cell type.

Chromatin data provide the strongest observational discriminator, although they cannot establish causality on their own. Generic stress can remodel stress-responsive chromatin without disrupting the elements that define neuronal subtype. Identity erosion more specifically predicts reduced accessibility across coordinated sets of subtype-defining enhancers, especially when accompanied by reduced identity-factor motif activity and weaker coupling between those factors and their predicted targets [33]. For this reason, single-cell ATAC-seq or multi-omic comparisons between vulnerable and resistant populations are more informative than transcriptomic drift alone [34]. The relevant signal need not involve every subtype-specific enhancer. A coordinated and reproducible loss across a functionally related subset may be more informative than numerous isolated accessibility changes.

Identity erosion should therefore be evaluated against healthy cells of the same subtype, relatively resistant populations under comparable pathology, and cells undergoing a matched reversible response. These comparisons should control for technical degradation, global transcriptional decline, and selective loss of the most affected cells. Testing this prediction will require graded manipulation of maintenance capacity across a range of stressor loads. The manipulation should span from partial to near-complete disruption of the targeted identity architecture, verified through measurements of identity regulons, terminal-effector modules, and identity-enhancer accessibility. To distinguish an identity-specific effect from the generic burden of perturbing an essential cellular regulator, each identity-targeted manipulation should be compared with a non-identity perturbation calibrated before stressor challenge to produce a comparable change in prespecified generic readouts, such as metabolic capacity, proteostatic load, global transcriptional output, or baseline viability, while leaving subtype-defining regulatory architecture substantially intact. The identity-targeted manipulation should shift the stressor dose–response or recovery trajectory beyond the shift produced by this burden-matched control. If no degree of verified architectural disruption produces such an additional shift, identity-maintenance failure is not supported as a determinant of vulnerability in that population. A single manipulation with no phenotypic effect would be uninformative because the system may remain above the proposed threshold.

Such burden matching will necessarily be approximate because no control perturbation will reproduce every consequence of disrupting a multifunctional maintenance regulator. The generic readouts used for calibration should therefore be prespecified and reported explicitly, and conclusions should be tested across more than one calibration criterion where feasible. A practical implementation could use a titrated perturbation of an unrelated, broadly expressed cellular regulator, delivered with the same vector, promoter, recombination strategy, and experimental timing as the identity-targeted manipulation, and calibrated to produce a comparable change in baseline viability or metabolic capacity while leaving the predefined subtype program substantially intact.

Observational measurements of this kind can identify candidate identity-maintenance failures and position them within disease progression, but they cannot establish mechanism by themselves. In observational datasets, the framework is supported only if identity-regulatory measurements explain vulnerability beyond pathology burden, anatomical exposure, technical quality, and conserved stress responses. If they add little after these factors are considered, identity erosion is more parsimoniously interpreted as a correlate of degeneration. The interventional criteria described above would then be required to determine whether a candidate maintenance program shifts the boundary between recovery and irreversible dysfunction. Box 2 translates these principles into a practical workflow.

**Box 2. Practical tests for distinguishing state remodeling from identity-maintenance failure**

1. Prespecify subtype boundaries, identity regulons, and terminal-effector modules from healthy reference data before examining disease samples.
2. Prespecify an operational composite across four domains: identity factor target coupling, identity regulon and terminal effector coherence, identity-enhancer accessibility or motif activity, and repression of excluded programs. Where all four domains are biologically applicable and technically measurable, require disruption in at least three, with each crossing an assay-specific effect-size threshold relative to the matched control distribution.
3. Test whether the results remain stable across biologically plausible clustering and annotation resolutions.
4. Compare vulnerable and resistant populations at matched disease stage, tissue context, pathology exposure, and cell quality.
5. Separate loss of subtype coherence from global transcriptional decline, RNA degradation, survivorship bias, or selective loss of the most affected cells.
6. Ask whether conserved stress-response modules explain vulnerability, or whether identity-regulatory erosion adds explanatory power.
7. Test whether identity transcription factors remain coupled to their predicted target genes.
8. Use chromatin or multi-omic data to determine whether coherent sets of subtype-defining enhancers and identity-factor motifs remain accessible.
9. Look for partial mixed-identity programs, including fragments of neighboring or sibling cell-type modules that are normally excluded.
10. In spatial data, ask whether vulnerable cells blur towards nearby related populations rather than towards arbitrary cell types, while testing whether those populations share comparable pathological and niche exposure.
11. Where experimentally possible, remove the initiating perturbation and test whether identity regulons, terminal-effector modules, and identity enhancers return to the matched recovered-control range or instead show hysteresis.
12. Include a non-identity control perturbation calibrated before stressor challenge to produce a comparable change in prespecified generic cellular-burden readouts, such as metabolic capacity, proteostatic load, global transcriptional output, or baseline viability, without coordinated erosion of subtype-defining architecture.
13. Use graded, bidirectional manipulations spanning partial to near-complete disruption or preservation of the targeted identity architecture, and test them across a range of stressor doses. Confirm the intended architectural effect by measuring identity regulons, terminal-effector modules, and identity-enhancer accessibility. Require the identity-targeted manipulation to shift vulnerability or recovery beyond the effect of the burden-matched non-identity control. If no degree of verified disruption produces such an additional shift, reject identity-maintenance failure as a determinant in that population. A single null manipulation is not decisive. Because matching will be approximate, report the readouts used for calibration and the residual differences between perturbations. Where fewer than four domains are measurable, label the classification provisional and report which domains remain untested.
14. Treat identity erosion seen only after broad terminal decline as a consequence of degeneration, not as evidence for the proposed mechanism.
15. Where manipulation of a maintenance node changes survival without preserving identity architecture, consider a parallel metabolic, trophic, or homeostatic explanation.

## Future research directions and applications

Selective vulnerability is usually framed as a problem of why particular neurons fail. That framing remains essential. It should, however, be paired with the reverse question: why do related neurons survive? As explored in this Essay, the answer may not be only that resistant neurons experience less stress or have more favorable physiology. In some populations, resistance may also depend on whether subtype-defining regulatory architecture remains functional as stress increases. Although further work is required to test the threshold framework presented above, we can already start to explore different research avenues that it could be applied to.

### Testing the role of tissue environmental factors

The identity-maintenance framework is cell-centered, but resistance is unlikely to be entirely cell intrinsic. Neurons maintain identity in a tissue environment shaped by astrocytes, oligodendrocytes, vasculature, extracellular matrix, peripheral signals, and microglia. These neighboring cells can influence trophic support, inflammatory tone, synaptic stability, metabolic substrate availability, and clearance of protein aggregates [35,36]. The resulting niche may affect selective vulnerability in at least two ways: by altering the stress imposed on a neuron or by altering the stability of the regulatory machinery through which that neuron responds. These possibilities will need to be distinguished experimentally.

Microglia are a useful example because they are not merely inflammatory effectors. They regulate development, synaptic maintenance, injury responses, and clearance functions in the adult central nervous system [35]. In neurodegeneration, disease-associated microglial states can reflect responses to tissue damage and may also modify the disease trajectory through TREM2- and APOE-related pathways [36]. For this framework, the relevant question is whether microglial or other niche states merely alter the stress burden or also affect the stability and recovery of neuronal identity-regulatory programs. An intervention that lowers pathology would constitute upstream protection. Evidence for niche-supported identity maintenance would require preservation of subtype-defining regulatory architecture at a comparable pathology burden.

Aging also intersects with resistance. Age is the major risk context for most neurodegenerative diseases, and aging affects proteostasis, mitochondrial function, DNA and RNA maintenance, inflammation, nutrient sensing, and chromatin state [37,38]. These changes may narrow the range over which neuronal responses remain reversible, bringing some populations closer to identity-maintenance failure before disease-specific pathology develops. Geroscience-inspired interventions could therefore shift the recovery threshold indirectly, but they should be described as identity-maintenance interventions only if they preserve subtype-defining regulatory coherence beyond their effects on generic stress or pathology.

### Therapeutic and clinical avenues

The clinical implication is not that clinicians should measure neuronal identity maintenance tomorrow. Rather, drug discovery and biomarker development may need to distinguish between two forms of protection. One protects the cell from acute stress. The other preserves the regulatory program that allows the cell to retain its subtype identity and recover after stress. These forms of protection may overlap, as the Nurr1 example suggests, but they are not conceptually identical. If identity-maintenance failure can become self-sustaining, treatment timing becomes part of the mechanism. The same intervention might restore a maintenance circuit before the transition but fail after the regulatory state has become self-reinforcing. Identity-regulatory biomarkers could then serve not only as correlates of damage, but also as indicators of whether vulnerable neurons remain on the recoverable side of that boundary.

For therapy, the most direct levers would be transcriptional or epigenomic nodes that maintain vulnerable neuronal identities. Nurr1 is an experimentally tractable maintenance node in dopamine neurons, but it should not yet be taken as proof that identity maintenance explains selective vulnerability. Comparable nodes must be identified and tested causally in other vulnerable neuronal populations. Direct manipulation of transcription factors or chromatin regulators may also carry risks because excessive or prolonged intervention could distort mature identity, activate inappropriate developmental programs, or alter multiple non-identity functions. Therapeutic strategies may therefore need to be subtype-restricted, graded, and time-limited rather than designed simply to maximize expression of a maintenance factor.

A more concrete indirect lever is trophic support. Nurr1 loss reduces Ret-dependent GDNF signaling, whereas adult Pitx3 loss is accompanied by early GDNF reduction in SNc neurons [24,31]. This convergence suggests that maintenance networks may support the trophic competence of vulnerable neurons. Trophic intervention would support the identity-maintenance mechanism specifically only if it preserved subtype-defining regulatory architecture rather than merely reducing cellular stress.

For biomarkers, identity-maintenance failure suggests that early disease should be assessed not only by cell loss or generic stress signatures but also by the preservation of subtype-defining modules. Human single-nucleus, spatial, and epigenomic datasets could be reanalyzed to determine whether resilient neurons preserve identity-regulatory coherence despite pathology. Such signatures are currently better viewed as research biomarkers and target-validation readouts than as clinically accessible measurements. Their value will depend on whether they predict resilience or slower progression after accounting for pathology burden, disease stage, anatomical location, conserved stress responses, and technical quality.

### A reanalysis and intervention agenda for Alzheimer’s disease and ALS

Alzheimer’s disease offers a rich setting for analysis because single-cell and single-nucleus datasets have identified vulnerable excitatory populations and disease-associated transcriptional states across the entorhinal cortex, hippocampus, and association cortex [39–41]. The identity-maintenance question is whether vulnerable neurons show erosion of subtype-defining regulons before generic terminal decline, and whether resilient neurons preserve those modules under comparable amyloid, tau, inflammatory, or metabolic burden. Identity regulons should be defined using healthy reference data rather than reconstructed from surviving diseased cells, and their explanatory value should be tested after accounting for pathology and conserved stress-response modules.

RORB has been identified as a marker of entorhinal excitatory populations that are preferentially depleted and selectively susceptible to neurofibrillary inclusions during Alzheimer’s disease progression [40]. The study did not establish that RORB maintains these neurons in adulthood or that loss of RORB activity causes their vulnerability. It nevertheless defines a concrete test: determining if the RORB-associated regulatory network is continuously required for adult entorhinal subtype identity, whether this network erodes before terminal decline, and if manipulating it changes vulnerability at matched tau burden. At present, this remains an observational and experimental hypothesis rather than an established mechanism in Alzheimer’s disease.

ALS poses a parallel question in motor neuron biology. Fast-fatigable motor neurons are selectively vulnerable to degeneration, whereas other motor neuron subtypes remain resistant for longer [5,6]. Classic explanations emphasize excitability, proteostasis, endoplasmic reticulum stress, neuromuscular junction stability, and axonal demand. Recent interventional evidence brings selector transcription factors directly into this discussion. Viral re-expression of the embryonic motor neuron programming factors ISL1 and LHX3 in postnatal motor neurons reactivated aspects of a youthful gene expression program and reduced disease-associated pathology and motor neuron degeneration in the *SOD1*^*G93A*^ mouse model [42]. This study provides important interventional evidence that lineage-regulatory activity can modify neuronal resilience, but it is not direct evidence for maintenance of the mature adult identity program. Endogenous *Isl1* and *Lhx3* expression declined sharply after their embryonic peak and ISL1 and LHX3 were undetectable by immunofluorescence in ChAT-positive L4-L5 ventral-horn motor neurons at postnatal day 45 in untreated mice. The intervention therefore reintroduced a developmental regulatory program that was not detectably active in the adult population examined. The result supports the related claim that redeployment of lineage selectors can improve stress buffering, while showing that resilience need not always arise from preserving the existing adult state.

This distinction also identifies a useful boundary condition. The motor neurons examined had reached mature postnatal states without detectable ISL1 or LHX3 immunoreactivity [42]. Their mature identity may therefore be maintained by other transcriptional or chromatin mechanisms, or selector-based maintenance in adulthood may not be the relevant determinant of resilience in this population. The next experiments should identify the endogenous adult maintenance circuitry of vulnerable and resistant motor neuron subtypes and test whether its manipulation shifts vulnerability independently of the developmental reprogramming induced by ISL1 and LHX3.

## Conclusions

In this Essay, I proposed that identity-maintenance capacity may act as a threshold-setting variable that can influence whether a stressed neuron returns to its previous subtype state or enters self-reinforcing regulatory decline. Nurr1 loss establishes that adult dopaminergic identity and function require continuous transcriptional maintenance. Differential dependence on Pitx3 shows that related neuronal populations can organize shared identity factors differently. PRC2 perturbation separates identity destabilization from cell death and reveals population-specific consequences of disrupting repressive maintenance. These findings make the mechanism experimentally tractable, but they do not resolve the confound between identity-maintenance fragility and established physiological liabilities.

The central test is not whether identity markers decline before neurons die. It will be whether a manipulation that demonstrably alters subtype-defining regulatory architecture also changes the stress level at which function fails and the capacity to recover after stress removal, beyond the change produced by a non-identity perturbation matched for generic cellular burden. If no degree of verified architectural disruption changes either trajectory beyond this control, identity-maintenance failure is not the operative mechanism in that population. Nonlinear and path-dependent recovery would provide more distinctive evidence for a self-reinforcing maintenance threshold.

If this framework holds, selective vulnerability should be studied not only as a catalogue of cellular liabilities, but also as a failure of active maintenance. The decisive experiments will separate maintenance capacity from classical physiological burden and test vulnerable and resistant populations across controlled stress and recovery trajectories. Whether preserving identity architecture shifts the boundary between reversible remodeling and irreversible dysfunction is the empirical question on which the framework will stand or fall.

## Abbreviations

ALS: amyotrophic lateral sclerosis
SNc: substantia nigra pars compacta
UPR: unfolded protein response
VTA: ventral tegmental area
